# Decrease in fMRI brain activation during working memory performed after sleeping under 10 lux light

**DOI:** 10.1038/srep36731

**Published:** 2016-11-09

**Authors:** Seung-Gul Kang, Ho-Kyoung Yoon, Chul-Hyun Cho, Soonwook Kwon, June Kang, Young-Min Park, Eunil Lee, Leen Kim, Heon-Jeong Lee

**Affiliations:** 1 Department of Psychiatry, Gil Medical Center, Gachon University, School of Medicine, Incheon, Korea; 2 Department of Psychiatry, Korea University College of Medicine, Seoul, Korea; 3 Department of Anatomy, Korea University College of Medicine, Seoul, Korea; 4 Department of Biomedical Science, Korea University College of Medicine, Seoul, Korea; 5 Department of Psychiatry, Ilsan Paik Hospital, Inje University College of Medicine, Goyang, Korea; 6 Department of Preventive Medicine, Korea University College of Medicine, Seoul, Korea

## Abstract

The aim of this study was to investigate the effect of exposure to dim light at night (dLAN) when sleeping on functional brain activation during a working-memory tasks. We conducted the brain functional magnetic resonance imaging (fMRI) analysis on 20 healthy male subjects. All participants slept in a polysomnography laboratory without light exposure on the first and second nights and under a dim-light condition of either 5 or 10 lux on the third night. The fMRI scanning was conducted during *n*-back tasks after second and third nights. Statistical parametric maps revealed less activation in the right inferior frontal gyrus (IFG) after exposure to 10-lux light. The brain activity in the right and left IFG areas decreased more during the 2-back task than during the 1- or 0-back task in the 10-lux group. The exposure to 5-lux light had no significant effect on brain activities. The exposure to dLAN might influence the brain function which is related to the cognition.

Nighttime light is now considered to be one of the fastest growing pollutants, and the invasion of artificial light into previously unlit areas is threatening the soundness of human health and sleep. Nighttime artificial lighting in cities is divided into three types: sky glow, trespass, and glow. Light trespass refers to unwanted direct lighting of an area, and it occurs when unwanted light spills over into another property or dwelling and causes sleep interference, negative influence on one’s well-being, and impaired visibility of evening star^1^.

Concerns about and distress from the ecological consequences of nighttime artificial light are increasing. Several studies have also shown that light pollution and shift work are tentative risk factors for cardiovascular disease, breast cancer, ovarian cancer, gastrointestinal disease, and metabolic syndrome^2^,^3^,^4^,^5^. However, systematic and well-controlled studies of the harmful effects of light pollution on humans are lacking.

Many countries have recently enacted laws and regulations for reducing light at night (LAN), many of which have been based on international guidelines, such as Commission Internationale de l’Eclairage (CIE), Illuminating Engineering Society of North America (IESNA), and Institution of Lighting Engineers (ILE)^6^. The upper brightness limits for light trespass into residential areas in cities are 2, 3, and 5 lux in the CIE, IESNA, and ILE guidelines, respectively^6^. South Korea recently adopted a regulation setting the upper limit for light trespass to 10 lux in city residential areas for practical reasons, but public demands for stricter regulations (i.e., 5 lux or dimmer) have increased since then. For the reason that no research has been made on the intensity of dim light at night (dLAN) which causes health problems including sleep disturbance, discussions and debates are in slow progress about setting relevant regulations. The authors reported that the exposure to the dLAN (5- and 10- lux) while sleeping was significantly associated with an increased duration of awake time after sleep onset, increased N1 (stage 1) sleep, and decreased N2 (stage 2) sleep in the previous study which was performed with the same study design^7^. However, the evidence of and biological studies on effects of dim light trespass on cognition, sleep, and health are still insufficient. For this reason, further scientific evidence and studies regarding the effects of dLAN (e.g., 5 vs 10 lux) on cognition and brain activation are necessary to ensure that reasonable regulations are formulated to protect against adverse effects of light trespass.

While several studies have investigated the effects of light exposure on cognitive function, alertness, electroencephalography changes, and brain function, these have been limited to the subjects of immediate waking and stimulation effects after light exposure^8^,^9^,^10^,^11^,^12^. There has been little research into changes in cognitive function and human brain activation after LAN exposure during sleep. In particular, no previous study has used functional magnetic resonance imaging (fMRI) to investigate changes in human brain activation after dLAN exposure during sleep.

This study investigated the effect of dLAN (5 vs 10 lux) when sleeping on the brain activation of healthy young men during a working-memory (WM) task (*n*-back task) using fMRI.

## Results

### Demographic data

The demographic data, clinical characteristics, and baseline *n*-back task and nocturnal polysomnography (NPSG) results are given in Table 1. The age, duration of education, *n*-back task results, and NPSG results at the baseline evaluation did not differ significantly between the 5- and 10-lux groups (Table 1). No group difference was found either in the difference of the working memory task performance performed in different task difficulties using the mixed-model analysis of variance (ANOVA) to investigate the presence of a group × task difficulty interaction.

### n-back task

Changes in the *n*-back task results between before and after the light exposure were analysed separately in the 5- and 10-lux groups. While the reaction time before and after the LAN was significantly different based on the 1 back task (*t* = 3.41, *p* = 0.007) and the 2 back task (*t* = 2.86, *p* = 0.017) of 5 lux group, the response accuracy was not different before and after the LAN. The reaction time as well as the response accuracy before and after the LAN did not show any difference based on all *n* back tasks of the 10 lux group, although the response accuracy on the 1 back task of 10 lux group tended to decline after the light exposure (*p* = 0.059).

**Figure 1:**
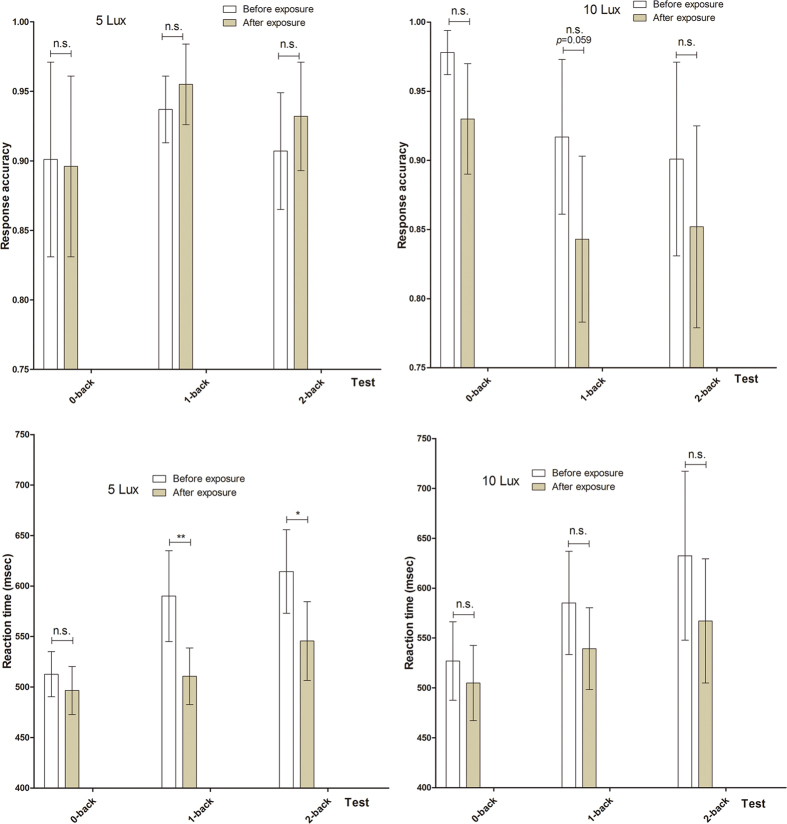
Response accuracies and reaction times for 0-, 1-, and 2-back tasks before and after exposure to LAN during sleep of patients allocated to 5- and 10-lux groups. Response accuracy (ratio) and reaction times (msec). Data are mean and standard error values. Abbreviation and mark: LAN, light at night; n.s., not significant; *p < 0.05; **p < 0.01.

With regard to the difference between the 5 lux group and the 10 lux group in their response accuracy and the reaction time before and after the LAN (Fig. 1), the response accuracy in the 1 back task after the LAN in the 10 lux group decreased more than that of the 5 lux group (*F* = 4.75, *p* = 0.043). The change of the response accuracy, however, showed no difference in 0 back and 2 back tasks between two groups, and the change of the reaction time to all *n* back tasks did not show any significant difference between two groups, either.

### fMRI findings

**Figure 2:**
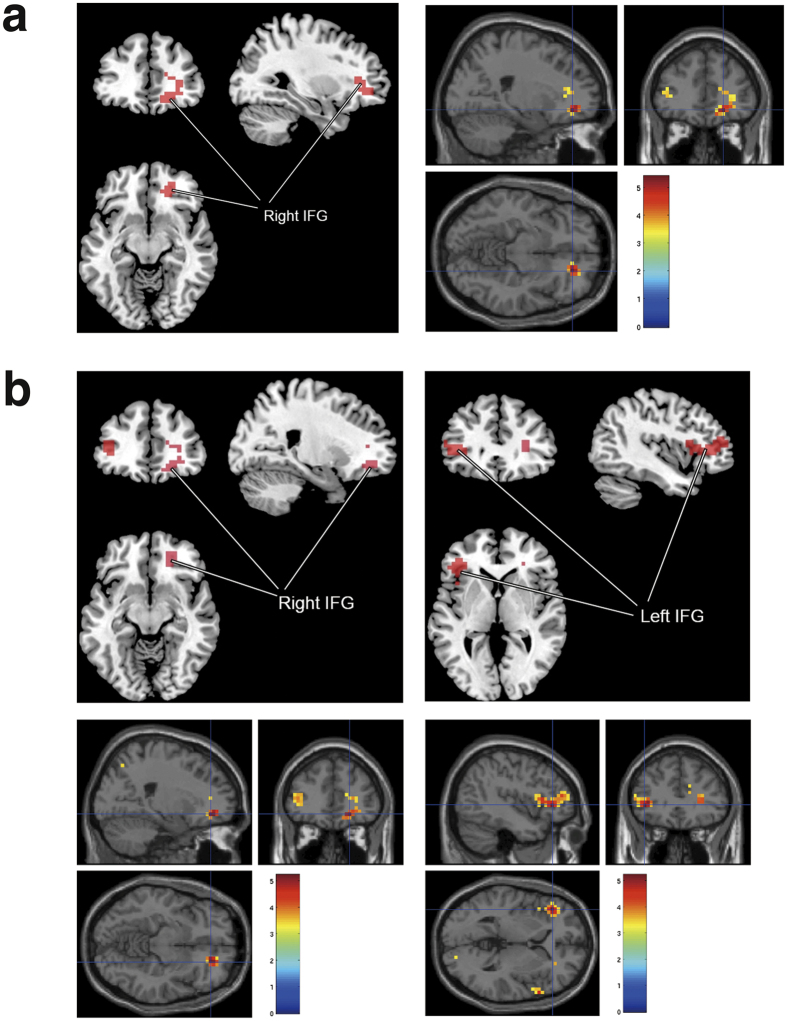
Decreases in fMRI activities in the IFG after exposure to 10-lux light. **(a)** Significant decreases in fMRI activities in the right IFG after exposure to 10-lux light for one night. Before exposure > after exposure. *P*_FWE-corr_ = 0.014. Statistical threshold of *p* < 0.05 (corrected) at the cluster level. **(b)** fMRI activities in the IFG after exposure to 10-lux light decreased more when performing a more difficult task (i.e., 2-back task compared to 1- or 0-back task). Before exposure >after exposure, 2-back task >1- or 0-back task. *P*_FWE-corr_ = 0.033 in right IFG. *P*_FWE-corr_ = 0.010 in left IFG. Statistical threshold of *p* < 0.05 (corrected) at the cluster level. Abbreviations: fMRI, functional magnetic resonance imaging; IFG, inferior frontal gyrus; FWE-corr, family-wise error corrected for multiple comparisons.

In the comparisons between pre- and postexposure to light of 10 lux for one night, the light exposure was associated with a decrease in brain activation. The statistical parametric mapping (SPM) of brain regions (paired *t* test; before vs after exposure to 10-lux light) revealed that the activation during the *n*-back task in the right inferior frontal gyrus (IFG) was significantly less after exposure than before exposure (*T* = 5.39, *p*_FWE-corr_ = 0.014; FWE-corr refers to the family-wise error corrected for multiple comparisons at cluster-level; *d* = 1.527; Fig. 2-a, Table 2). The coordinates for maximal activation in these clusters were (*x*, *y*, *z*) = (20, 40, −10) of the right IFG and a post-hoc power analysis indicated the power of 0.978. However, the exposure to 5-lux light did not decrease brain activation.

The fMRI activity in the right and left IFG decreased more when performing the 2-back task than the 1- and 0-back task (2-back task >0- and 1-back task) after exposure to 10-lux light for one night (right IFG, *T* = 5.21, *p*_FWE-corr_ = 0.033; left IFG, *T* = 5.14, *p*_FWE-corr_ = 0.010; Fig. 2-b, Table 2). However, no such pattern was found in the 5-lux group.

**Figure 3:**
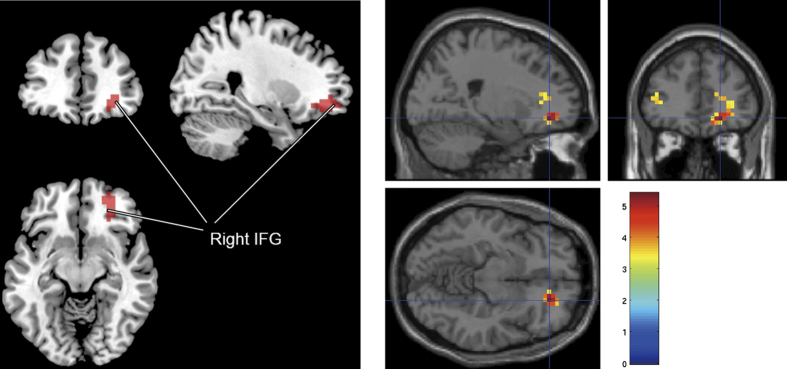
fMRI activities in the right IFG decreased more in 10-lux group than in 5-lux group. Comparison between 5- and 10-lux groups (uncorrected). 10-lux exposure >5-lux exposure, Activation decrease. *P*_uncorrected_ = 0.017, *P*_FWE-corr_ = 0.083. Repeated-measures analysis of variance was used for the statistical analysis. Abbreviations: fMRI, functional magnetic resonance imaging; IFG, inferior frontal gyrus.

We determined whether the difference in the decrease between the groups (10 vs 5 lux) was significant using the mixed-model ANOVA to investigate the presence of a significant group × time interaction. The fMRI activity in the right IFG decreased more in the 10-lux group than in the 5-lux group during the *n*-back task (*T* = 4.04, *p*_uncorrected_ = 0.017, *p*_FWE-corr_ = 0.083; Fig. 3, Table 2).

In the 10-lux group, there was no significant correlation of the blood oxygen level-dependent (BOLD) signal change in the right IFG after dLAN with the change of sleep efficiency (*p* = 0.332) and the wake time after sleep onset (*p* = 0.951). In all participants, the correlation of the BOLD signal change with the change in sleep efficiency (*p* = 0.792) and the wake time after sleep onset (*p* = 0.937) was not significant, either.

## Discussion

Many recent studies have investigated the effects of light pollution and light trespass on humans, but there has been no previous study using fMRI researching into the effect of the dLAN on the WM. To the best of our knowledge, this is the first report on a decrease in brain activation detected by fMRI during an *n*-back task (a WM test) after exposure to dim light (10 lux) while sleeping. This study is meaningful because it is the first to scientifically identify the effect of the dLAN on human brain function and cognition. It is noteworthy that the brain activation was altered after only a single night of light exposure. This suggests that the chronic exposure to the LAN for many nights might have caused more pronounced effects on the brain and cognition.

Comparing the working memory results and fMRI results of any *n* back task, the response accuracy of 10 lux group in 1 back task after the LAN decreased more than that of the 5 lux group, which is in line with the decline of the brain activation in fMRI result. The interesting finding in the 10 lux group, however, was the discrepancy between the *n*-back task and fMRI results. The decrease of the brain activation in fMRI in the frontal lobe without significant finding in the *n*-back task of 10 lux group suggests that the absence of evidence of subjective or objective cognitive dysfunction does not necessarily mean that the brain is functioning normally. This indicates that certain exposure to dim light might influence brain function for cognition even if there is no significant impairment in subjective symptoms (or even in an objective neurocognitive function test). The reaction time at 1 back and 2 back tasks after the LAN decreased in 5 lux group while that of 10 lux group showed no significant difference. This can be deemed as evidence that decrease of BOLD signal in 10 lux group at fMRI is not attributable to the learning effect. If we assume that the decrease in BOLD signal is attributable to the less cognitive effort by learning effect, the decrease in BOLD signal should have been found rather in 5 lux group.

We observed significant decreases in fMRI activity in the right IFG after exposure to 10-lux light. The IFG is believed to play an executive role in the WM^13^,^14^. The decrease in the activation of the IFG during the *n*-back task after the light exposure may suggest the deterioration of brain function. The decreases in fMRI activity in both frontal gyri were more significant when performing the 2-back task than performing simpler tasks after exposure to 10-lux light. Past researches have also found a linear relationship between the task load during the *n*-back WM test and the cerebral blood flow in fMRI; specifically, an increased task load intensifies the decompensation of frontal lobe function^15^.

The decrease in brain activation might be attributable to the poor quality of sleep during exposure to the dLAN. In our previous study where we applied the same study design as this study^7^, we concluded that the exposure to dLAN during sleep caused an increase in the frequency of arousal and the amount of shallow sleep, which we thought attributable to a change in melatonin secretion^7^. Ikeda showed that continuous exposure to dim light (0.5~1 lux) caused changes in the sleep and body-temperature patterns of adult rats: the time spent in NREM sleep decreased after the first night of dim light exposure, while the number of REM sleep episodes increased (i.e., greater fragmentation of REM sleep)^16^. They concluded that the amount of sleep and its structure are vulnerable to both short-term and prolonged exposures to dim light. Another study exposed eight healthy male volunteers to an ordinary room light (~180 lux), and found that this has reset the circadian melatonin and cortisol rhythm, countering an earlier study that indicated the phase shifting of the suprachiasmatic nucleus could be induced by exposure to light of moderate-intensity (i.e., 500 lux)^17^,^18^.

Previous fMRI studies have found that sleep deprivation changed certain brain function during the WM test; however, the results have somewhat varied^19^. The most consistent finding of neuroimaging studies is that the sleep deprivation decreases brain activation in response to WM tests in the frontal cortex^20^,^21^,^22^ and the posterior (parietal, occipital, and temporal) cortices^21^,^23^. On the other hand, other studies have found that the activation in thalamic nuclei, anterior cingulated cortex, and prefrontal area increased after sleep loss, which was interpreted as a compensation phenomenon for the effects of sleep deprivation^20^,^21^,^23^.

In the correlation analysis, no significant correlation was found between the change of the BOLD signal in IFG and the sleep measurements (i.e., sleep efficiency and wake after sleep onset). LAN exposure might directly induce cognitive dysfunction, rather than indirectly via sleep disturbance. One previous study has found that the irregular light schedule directly impaired both learning ability and mood via melanopsin-expressing neurons in mice^24^. Interestingly, despite their indication of normal circadian rhythm and sleep structure, the subject mice showed impaired long-term potentiation of the hippocampus and learning in the presence of aberrant light exposure^24^. This cognitive impairment was attributable to the intrinsically photosensitive retinal ganglion cells (ipRGCs), which project not only to hypothalamic and preoptic areas but also to the limbic regions such as the lateral habenula and medial amygdala^24^. Bedrosian *et al.* argued that the dLAN might directly influence the brain function via a process related to the ipRGCs, and an analysis of behaviour and hippocampal morphology showed that exposing hamsters to dLAN (5 lux) for 4 weeks altered their neuronal structure and generated a depressive response^25^. Fonken *et al.* reported that exposure to 5-lux LAN for 3 weeks impaired the cognition (learning and memory in the Barnes maze test) and provoked depression-like responses in a diurnal rodent^26^. They also showed a reduced dendritic length in the dentate gyrus and basilar CA1 area of the hippocampus neurons in the rodent after exposure to dLAN. Bedrosian *et al.* explained that these changes in the hippocampus resulted from decreases in BDNF expression and highly plastic dendritic spines induced by environmental changes^25^,^27^,^28^.

This study has several limitations. Firstly, the sample size was relatively small and this could result in lower statistical power in the neuroimaging analyses. Secondly, we did not have a control subject group who spent 3 nights without any dLAN. Thirdly, we could not control the previously accumulated potential LAN effect prior to the experiment. Further studies with larger samples and control subject group are therefore warranted.

The results of this study suggest that exposure to LAN during sleep reduces brain activation, which has major implications for further follow-up studies and the development of governmental policies. The effect of 5-lux LAN exposure on sleep and cognition should be investigated further, even though the reduction in brain activation was not statistically significant in the 5-lux group in the present study. Moreover, effects of long-term exposure to dim light while sleeping on brain activation, cognitive function, sleep problems, and brain morphology will also need to be further investigated in larger scale studies.

## Methods

### Subjects

We recruited young male healthy volunteers who had no history of sleep, psychiatric, or major medical disorders. The subjects were aged 19–29 years, and each provided informed written consent prior to the enrollment in this study. The study protocol was approved by the Institutional Review Board (IRB) of Korea University Anam Hospital (IRB number: ED 12261) and was conducted in accordance with the Declaration of Helsinki. We enrolled only young healthy male subjects in order to better eliminate the impact of other factors such as age, sex, and health-related confounders.

The inclusion criteria were as follows: (i) right handedness, (ii) no current or past sleep disorders or neurocognitive disorders, (iii) no current or past medical, neurological, or psychiatric illness that may affect brain function or cognitive function, and (iv) have not taken any neurotropic or psychotropic medications for their life time. The exclusion criteria were as follows: (i) suspected of having sleep–wake cycle irregularity or circadian rhythm sleep disorders in actigraphy, (ii) suspected of having any sleep disorders in Night 1 NPSG (e.g., SE below 90%, apnea-hypopnea index >5, periodic limb movement during sleep >15, or presence of REM sleep without atonia), (iii) body mass index of >30 kg/m^2^, (iv) contraindication for 3T MRI (such as claustrophobia and pacemaker), and (v) structural abnormality in brain MRI.

After the initial screening of subjects’ sleeping conditions, physical health, and psychiatric health based on questionnaires, 35 subjects were interviewed face-to-face in order to evaluate their eligibility for the study by a board-certified psychiatrist (H.J.L.) majoring in sleep medicine using the semi-structured interview of DSM-IV-TR. Based on such interview, we excluded five subjects who were suspected to have a specific sleep disorder, such as obstructive sleep apnea, aberrant sleep–wake cycle, or obesity.

The participants were randomly divided into two groups and exposed to different light-intensity: 5 and 10 lux. For 1 week prior to the study, 30 participants wore an actigraph (Actiwatch-L, Mini Mitter, Bend, OR) on their non-dominant wrist in order for us to identify their sleep–wake cycles and screen the potential sleep–wake disorders and excessive LAN. We reviewed the actigraphic data and the information of the light meter for all of the participants’ 1-week sleep–wake cycles, and excluded two participants whose cycles were disturbed due to being irregular or the presence of suspected delayed-sleep-phase syndrome. One participant withdrew the consent to participate in the middle of the study. After scoring and staging the NPSG results of all participants, we further excluded four participants with a sleep efficiency of <90% or an apnea-hypopnea index of >5 in NPSG.

Finally, 23 subjects participated in our study: 11 and 12 subjects in the 5- and 10-lux groups, respectively. The fMRI analysis included 20 subjects (11 and 9 in the 5- and 10-lux groups, respectively), since 3 participants were excluded due to missing data caused by a task-button recognition error.

### Study design

**Figure 4:**
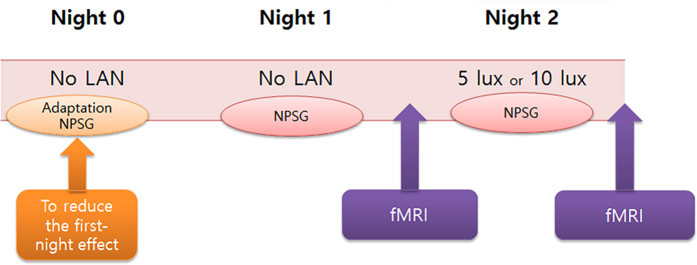
Schematic of the LAN intervention procedure. To reduce the first-night effect, we have applied an additional nocturnal NPSG session on the first night (Night 0) before performing the main study on the second and third nights (Nights 1 and 2). After Nights 1 and 2, participants underwent fMRI while performing *n*-back tasks (0-, 1-, and 2-back tasks). Abbreviations: LAN, light at night; NPSG, nocturnal polysomnography; fMRI, functional magnetic resonance imaging.

All of the participants were requested to sleep and be awake according to standard time schedule during a period from one week before the experiment through the end of this study, and were prohibited from napping during the daytime and consuming medicines, caffeine beverages, and alcohol. Participants were also asked to keep their routine activity and the usual brightness of lighting. NPSG was conducted for three consecutive nights (Fig. 4). During each NPSG session, the lights were turned off at 11:00 p.m. and turned on at 7:00 a.m. After the lights were turned off, participants were prohibited on the use of smartphones during the entire NPSG session. All participants underwent adaptation NPSG on the first night (Night 0) to adapt to the NPSG laboratory and to minimise the first-night effect of the NPSG. Subjects were instructed to sleep in the NPSG room without any light exposure on the second night (Night 1) and under a dim light condition of 5 or 10 lux on the third night (Night 2). The participants were asked to sleep in the supine position as much as possible and not to hide their face with a blanket. After the second and third nights, participants underwent fMRI while performing *n*-back tasks (0-, 1-, and 2-back tasks) in order to evaluate their cognitive function (Fig. 4).

For the whole period of Night 2, a light box producing a dim light with a daylight ‘cool white’ colour was placed on the upper part of the wall opposite to participant’s head. The box contained a light source comprising wide-spectrum light-emitting diodes with an encompassing wide wavelength (dom λ: 501.4 nm, peak λ: 463.6 nm, centre λ: 467.6 nm, centroid λ: 554.3 nm, Correlated Colour Temperature: 5779.1 K, General Colour Rendering index: 90). The luminous intensity was set to 5 or 10 lux according to the study group. Light sources were enclosed in a box, approximately 11.8 × 11.8 × 1.3 inches, with a light diffuser panel on the front. The initial light box setup was installed by an illumination expert who is affiliated with the Korea Institute of Lighting Technology. The luminous intensity was checked by an illuminometer (ANA-F11, Tokyo Photo, Japan) at the level of the participant’s eyes while supine. Participants were not informed whether the dLAN was 5 or 10 lux.

### Polysomnography

The quality and quantity of subjects’ sleep were measured by NPSG for three consecutive nights (i.e., Nights 0, 1, and 2). Standard NPSG recordings were obtained using the Embla digital polysomnography and Somnologica software (Broomfield, CO, USA). The recording montage, electrode, and sensors of general parameters (i.e., electroencephalogram, electrooculogram, electromyogram, airflow signals, respiratory-effort signals, oxygen saturation, body position, and electrocardiogram) were applied according to the technical specifications recommended by the American Academy of Sleep Medicine (AASM)^29^. The polysomnography recordings were analysed on a computer monitor, and sleep stages and events were scored visually by a single well-trained polysomnography technologist based on the criteria of the AASM^29^, and all of the polysomnography data were confirmed by a sleep-specialist medical doctor (H.J.L.). Such NPSG findings on these subjects have been reported previously^7^.

### Performing n back task during functional scanning

**Figure 5:**
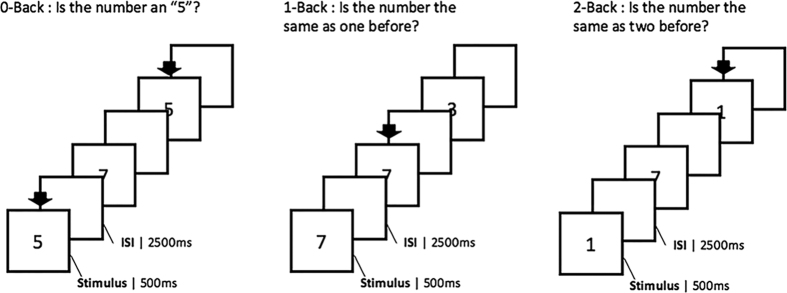
Sample trials of *n*-back tasks. The actual cues for n-back tasks were displayed in Korean. Abbreviation: ISI, interstimulus interval.

Participants performed a verbal version of the *n*-back task, which is a classical test of the WM. They were asked to monitor a series of stimuli and to respond whenever a presented stimulus (henceforth the “target” stimulus) was the same as the one previously presented *n* trials, where *n* is a pre-specified integer, usually 1, 2, or 3. Participants underwent one scanning session which is composed by six functional runs, each of which lasts 4 minutes and 47 seconds. The presentation of the stimuli was performed in a blocked design. Pre-generated random sequences of letters designed to equally distribute the targets and conditions in each runs were used^30^. The adopted event-related design was used to prevent the habituation to each condition (*n*-back conditions). Each condition (0-back, 1-back, 2-back) was equally distributed in each run. Each block was separated by a baseline control block lasting 16 seconds. Stimuli continuously appeared and the task required participants to temporarily store each stimulus in their memory for evaluation, and to discard it before the appearance of the next one. For this, three different conditions were used that varied the WM load incrementally from zero to two items. Each item was presented for 500 msec and the inter-stimulus interval was for 2500 msec. In the 0-back condition, participants responded to a single pre-specified target letter; in the 1-back condition, the target was any letter identical to the one immediately preceding it (i.e., one trial back); and in the 2-back condition, the target was any letter that was identical to the one presented two trials back. In this manner, the WM load (storage and manipulation demands) increased incrementally from the 0-back to the 2-back task (Fig. 5).

The stimuli were presented using Presentation 11.0 software (Neurobehavioral Systems, Albany, NY, USA). Responses of participants were collected using same software through fibre optic response pad (Current Designs, Philadelphia, PA).

### fMRI image acquisition

Anatomical T1-weighted magnetic resonance (MR) images were acquired at Korea University Brain Imaging Center, using a 3-tesla scanner (Tim Trio, Siemens, Erlangen, Germany) which had a 32-channel sense head coil (sense reduction factor = 2). An fMRI scanning was performed during *n* back task for each and every participant between 9:30 a.m. and 10:30 a.m. after PSG and we made sure that the MRI room used for all participants had the same light condition of 150 lux with cool-white colour. Functional images were acquired using gradient echo planar images with BOLD contrast and the following parameters: TR = 2000 msec, TE = 30 msec, flip angle = 90 degrees, field of view = 240 × 240 mm^2^, slice thickness = 4 mm, and 36 interleaved slices parallel to the anterior-commissure–posterior-commissure line covering the whole brain. Each scanning sequence comprised 164 sequential volumes. After the functional scanning, a high-resolution T1-weighted anatomical scan was performed on each participant with the following parameters: three-dimensional spoiled gradient echo sequence, 162 slices, TR = 1900 msec, TE = 2.32 msec, slice thickness = 1 mm, and in-plane resolution = 1 × 1 mm^2^.

### Data analysis: clinical and cognitive measures

The behavioral performance in the *n*-back task was assessed as the ratio of accurate responses and the average time to react with correct responses. The effects of LAN exposure on the 0-, 1-, and 2-back conditions were analysed based on the reaction time and response accuracy for patients allocated to 5- or 10-lux group. We compared the change in subjects’ performance after the light exposure in both 5- and 10-lux groups using either the paired *t* test or Wilcoxon signed-rank test and also compared differences in the exposure effect between the two groups using repeated-measures ANOVA. In case where the variance of differences in levels was not equal, the repeated-measures ANOVA was calculated using the Greenhouse and Geisser correction. All analyses were performed using SPSS for Windows, and the cutoff for statistical significance was set as *p* = 0.05.

### Image preprocessing and statistical analysis of fMRI data

Image processing and statistical analysis of imaging were performed using SPM version 8 (Institute of Neurology, London, UK) implemented in MATLAB (MathWorks, Sherborn, MA, USA). Before the image processing, board-certified neuroradiologists reviewed the structural MR images to check if there is any structural abnormality in their brain images. The first five volumes (obtained during 10 sec) of every run were discarded in order to avoid T1 equilibration effects, and all volumes were spatially realigned. The acquired images were spatially normalised to the Montreal Neurological Institute MNI152 stereotactic standard brain template according to the 12-parameter affine transformation and 16 nonlinear iterations^31^. The realigned and unwarped T2*-weighted volumes were spatially transformed and resampled in 3 × 3 × 3 mm^3^ voxels after normalisation. All functional volumes went through a spatial smoothing with an 8-mm full-width half-maximum isotropic Gaussian kernel in order to reduce the intersubject variability.

Due to the exploratory nature of this study, we adopted the whole-brain analyses instead of the region of interest (ROI) analysis. Statistical maps were generated using a random-effect model implemented through a two-level procedure. The first level of the analysis identified participant-specific task-related activations at the baseline and after dLAN exposure, by using a factorial model consisting of three active conditions (0-, 1-, and 2-back tasks). The second level of the analysis applied separate ANOVAs within SPM to contrasts of (i) 0-, 1-, and 2-back tasks >rest and (ii) 2-back task >0- and 1-back task. In addition to these, the data were tested for the exposure group (5 and 10 lux) × time (before and after exposure) interactions (the *difference* of *differences* in before vs after exposure under 5 and 10 lux) in a mixed-model repeated-measures ANOVA within SPM. Generic activations were identified in contrasts of (i) 0-, 1-, and 2-back task >rest and (ii) 2-back task >0- and 1-back task, using one-sample *t* test collapsing baseline data that were set for both groups. The resulting before- and after-exposure contrast values were subtracted (before *minus* after) in order to obtain the amount of *reduction* in cerebral activity from before- to after-exposure in any given region. Specifically, a positive number represents a decrease in activation. We then performed the correlation analysis between mean BOLD signal change in the cluster with a significant decrease in the activation and the change in sleep variables (sleep efficiency and wake time after sleep onset). Data were analyzed using voxel by voxel uncorrected threshold of *p* = 0.001. Then we selected only the clusters with a probability of activation of *p* < 0.05 after FWE correction for each cluster (extent threshold) unless otherwise indicated. A post-hoc power analysis was conducted with G*Power software to determine the retrospective power of the key finding of this study^32^.

## Additional Information

**How to cite this article**: Kang, S.-G. *et al.* Decrease in fMRI brain activation during working memory performed after sleeping under 10 lux light. *Sci. Rep.* **6**, 36731; doi: 10.1038/srep36731 (2016).

**Publisher’s note**: Springer Nature remains neutral with regard to jurisdictional claims in published maps and institutional affiliations.

## Figures and Tables

**Table 1:** Differences in demographic characteristics and baseline (i.e. prior light at night) *n*-back task and polysomnography results between 5- and 10-lux groups.

	5 Lux (*n* = 11)	10 Lux (*n* = 9)	Statistics
Duration of education (years)	13.1 ± 1.4	14.3 ± 1.7	*t* = −1.790, *p* = 0.090
*n*-back task results (baseline)			
RT (0-back task)	512.7 ± 74.1	526.9 ± 118.1	*t* = −0.329, *p* = 0.746
RT (1-back task)	590.0 ± 149.2	585.2 ± 155.2	*t* = 0.071, *p* = 0.944
RT (2-back task)	614.4 ± 137.4	632.5 ± 254.3	*t* = −0.191, *p* = 0.851
RAR (0-back task)	0.901 ± 0.233	0.978 ± 0.047	*t* = −0.964, *p* = 0.348
RAR (1-back task)	0.937 ± 0.079	0.917 ± 0.168	*t* = 0.355, *p* = 0.727
RAR (2-back task)	0.907 ± 0.140	0.901 ± 0.210	*t* = 0.068, *p* = 0.947
Polysomnography results (baseline)			
Sleep efficiency (%)	96.5 ± 1.7	97.6 ± 1.2	*t* = −1.651, *p* = 0.116
Sleep latency (min)	10.6 ± 8.4	12.9 ± 23.3	*t* = 0.980, *p* = 0.340
Sleep stage N3 (%)	18.6 ± 8.7	18.6 ± 6.0	*t* = −0.005, *p* = 0.996
Sleep stage REM (%)	21.5 ± 5.0	18.8 ± 4.1	*t* = 1.293, *p* = 0.213
AHI (*n*/h)	1.27 ± 1.26	1.17 ± 0.84	*t* = 0.216, *p* = 0.831
Arousal index (*n*/h)	12.4 ± 4.9	11.4 ± 3.1	*t* = 0.556, *p* = 0.585

Abbreviations: RT, reaction time (ms); RAR, response accuracy rate (ratio); REM, rapid eye movement; AHI, apnea-hypopnea index

Data are mean ± SD values.

Variables were analyzed using the independent *t* test.

**Table 2:** Decrease of brain activation after light exposure for one night.

Activation cluster	*K*	T-value (voxel level)	*p* value	MNI152 (mm)		
				*x*	*y*	*z*
**Before >after 10-lux light**						
Task total						
Inferior frontal gyrus	71	5.39	0.014	20	40	−10
2-back task >1-/0-back task						
Inferior frontal gyrus	56	5.21	0.033	22	40	−10
	77	5.14	0.010	−42	32	2
**Before >after light exposure**	**10 lux > 5 lux**					
Inferior frontal gyrus	43	4.04	0.017* (0.083)	26	40	−10

Abbreviation: MNI152, Montreal Neurologic Institute stereotactic standard brain template.

*K* = cluster extension in number of voxels (2 × 2 × 2 mm^3^).

*p*: corrected value (family-wise error corrected for multiple comparisons) unless indicated otherwise.

*Uncorrected *p* value for multiple comparisons.
